# The influence of early familial adversity on adolescent risk behaviors and mental health: Stability and transition in family adversity profiles in a cohort sample

**DOI:** 10.1017/S0954579419000191

**Published:** 2020-05

**Authors:** Ruth Wadman, Rachel M. Hiller, Michelle C. St Clair

**Affiliations:** 1 Department of Health Sciences, University of York, York, UK; 2 Department of Psychology, University of Bath, Bath, UK

**Keywords:** adolescence, early adversity, longitudinal cohort, mental health, victimization

## Abstract

Although familial adversity is associated with poorer outcomes in childhood and adulthood, little research has looked at the influence of stability or transition between distinct familial adversity subgroups or the impact in adolescence. Using data from the 9-month, 3-, 5-, and 14-year time waves of the Millennium Cohort Study (*n* > 18,000), we used latent class analysis to identify distinct classes of early familial adversity (marital instability/conflict, “suboptimal” parenting, economic disadvantage, and parental mental health problems) and the impact of these adversity classes on adolescent (a) mental health (including self-harm), (b) risk taking, (c) criminality, and (d) victimization. Four profiles were identified largely differing on economic hardship, family composition, and parental conflict. Across the first three time points, 72% of the sample remained stable, with the remainder transitioning between classes. Adolescents in the higher risk groups (particularly categorized by economic hardship or high parental conflict) had poorer outcomes in adolescence. Transitioning to a higher adversity group at any time in the first 5 years was associated with poorer outcomes but was particularly pronounced when the transition occurred when the child was under 3 years. These findings demonstrate the broad consequences of early familial adversity and the need for targeted early support for at-risk families.

Early adversity is a well-established risk factor for poor emotional adjustment and mental health outcomes later in life (Anda et al., 2002; Chapman et al., 2004; Dube et al., 2001; Dube, Anda, Felitti, Edwards, & Croft, 2002). At the most extreme end, child abuse and maltreatment predicts multiple problem outcomes across the life span, including poor mental health and risk behaviors such as alcohol and drug use and criminality (e.g., Kaplow & Widom, 2007; Norman et al., 2012; Thornberry, Ireland, & Smith, 2001). Beyond this, a growing body of research has examined the detrimental effect of other adversity types experienced within families on outcomes across childhood, including maladaptive family functioning and conflict (e.g., Kessler, Berglund, Demler, Jin, & Walters, 2005; Rhoades, 2008), maternal mental health (e.g.. Halligan, Murray, Martins, & Cooper, 2007), suboptimal parenting practices (e.g., harsh discipline; Flouri & Midouhas, 2017; Flouri, Midouhas, Joshi, & Tzavidis, 2015), and family economic stress (e.g., Masarik & Conger, 2017). Behavioral difficulties, psychopathology, and mental health problems are typically examined as outcomes of early childhood family adversity. For example, strong associations have been found between childhood adversity and depressive symptoms, antisocial behavior, and drug use, during the transition to adulthood (Schilling, Aseltine, & Gore, 2007). However, less is known, particularly outside of the United States, about early risk factors for more functional markers, such as the impact of adversity on criminality or broader risk-taking behavior (Bellis, Lowey, Leckenby, Hughes, & Harrison, 2014), or about the role of age of exposure to adversity, including whether there are developmentally more sensitive periods for exposure (though see Dunn et al., 2011). Further understanding the impact of early adversity, including the role of age of exposure to adversity, and in particular, the impact of early adversity on adolescent outcomes, remains an important area of investigation, with implications for early intervention for at-risk groups.

Early adverse life events and experiences generally do not occur in isolation. Instead, they are common and interrelated. The importance of the cumulative effect of multiple adverse childhood experiences on mental health outcomes is increasingly recognized (e.g., Hughes et al., 2017; Hughes, Lowey, Quigg, & Bellis, 2016). For example, co-occurring multiple childhood adversities (e.g., maladaptive family functioning, parental mental illness, childhood abuse, and neglect) have been found to have predictive and subadditive associations with both child-onset and adult psychopathology, with little specificity apparent across disorders (Green et al., 2010; Kessler et al., 2010). Cumulative early adversity risk factors measured across early childhood (<7 years) in the UK Avon Longitudinal cohort (including maternal psychopathology, single-parent household, being taken into care, abuse, and economic disadvantage) predicted preteen internalizing and externalizing behavior outcomes at age 11 (Slopen, Koenen, & Kubzansky, 2014). Furthermore, a New Zealand cohort study examining childhood predictors of adolescent suicidal behavior found that the risk of suicidality depended on accumulative exposure to social, family, and mental health adversity factors: those at greatest risk of suicidal behavior were young people whose family life was characterized by socioeconomic adversity, marital disruption, poor parent–child attachment, and exposure to sexual abuse (Fergusson, Woodward, & Horwood, 2000). Childhood adversities (assessed through retrospective reporting of stressful life events) have also been found to have a cumulative impact on drug and alcohol use in adolescence and early adulthood, with both recent and early adversity being significantly and independently associated with increased risk for drug and alcohol use (Lloyd & Turner, 2008).

Patterns of adversity (beyond simple quantification of adversity risk factors), and the issue of stability versus change, or transition, in early family adversity experienced across time has received less research attention (Dunn et al., 2011). It remains unclear whether families do regularly transition in and out of adversity (or whether familial adversity is largely stable) or whether such transitions can act to either protect or exacerbate later poor outcomes. Such information is important for informing early interventions for at-risk groups, as well as how timings of interventions may promote the best outcomes. To address these gaps in the literature, the first aim of this study was to use latent class analyses to characterize indices of early familial adversity (based on levels of economic stress, familial conflict, suboptimal parenting practices, and family structure) during a child's early development (between 9 months and 5 years) and to identify whether individuals move between classes over this time frame (i.e., transition away from or toward adversity). This approach permitted the identification of latent early family adversity subgroups (i.e., from lower to higher adversity) and movement between latent subgroups across early childhood, allowing patterns of stability and transition between risk groupings to be examined. The second aim of this study was to use these classes to explore how exposure to adversity in the preschool years (higher/lower risk groups) was associated with key markers of mental health (depression, self-esteem, and self-harm), risk behavior (substance misuse and early sexual behavior), criminal victimization, and criminal behaviors (antisocial behavior and criminal acts, and contact with police) during early adolescence. As a third aim, we also examined whether early transitions into, or out of, higher risk groups may protect from, or promote, later poor outcomes. To explore this, we have used the Millennium Cohort Study, a cohort study of approximately 19,000 UK children and parents. We have specifically utilized data collected in the first three waves, when the child was 9 months, 3 years and 5 years old, with outcomes assessed at 14 years of age.

Markers of risk were identified based on theory and the literature on early familial adversity, and included family structure (presence/absence of biological and nonbiological parents), parental conflict and partner use of force, suboptimal parenting (poor attachment/harsh discipline), economic disadvantage (including homelessness), and maternal depression. Previous research using cohort data have shown that single parenthood is a risk factor for poorer parent ratings of their child's adjustment at age 4, even after controlling for socioeconomic factors and parental depression (Dunn et al., 1998). Parental conflict has also been associated with internalizing and externalizing problems in childhood (Rhoades, 2008), and maladaptive or suboptimal parenting is associated with an increased risk of suicidal behavior in late adolescence (Johnson et al., 2002). Economic disadvantage has also previously been associated with poorer child adjustment, possibly via negative influences on parenting characteristics such as increased interparental conflict (Conger et al., 1991; Masarik & Conger, 2017). Lower socioeconomic status in childhood is related to a higher risk of major depression in adults (Gilman, Kawachi, Fitzmaurice, & Buka, 2002), while economic hardship during adolescence is associated with problem drinking, mediated through maternal psychological distress, parenting behaviors, and adolescent externalizing problems (Hardaway & Cornelius, 2014). The pervasive stress that comes with economic hardship has also been linked to suboptimal parenting practices, including increased use of harsh discipline (Neppl, Senia, & Donnellan, 2016). In turn, harsher parenting practices have been associated with increased child emotional and conduct problems (e.g., Flouri & Midouhas, 2017). Finally, maternal (or primary caregiver) mental health was also of interest as a key childhood stressor linked to adverse child outcomes, given evidence that it is a risk factor, albeit of small effect, for internalizing and externalizing difficulties in children (Goodman et al., 2011).

While early adversity is an established risk factor for poor outcomes, the ongoing impact on familial adversity in the preschool years on adolescent outcomes remains less well established. Developing a stronger evidence base for the impact of adversity on adolescents is particularly crucial, given adolescence is a time of marked increase in risk-taking behaviors (Rhoades, 2008) and the development of many common mental health problems (Jones, 2013). Our focus on cohort data at 14 years of age also means many of the outcomes are measured via young person report, reducing issues of single-informant bias, where parents report on adversity, their own mental health, and the child's mental health. Beyond the focus on adolescence, much of the literature is also either cross-sectional or relies on retrospective reporting of adversity, with fewer longitudinal studies examining risk behaviors in teenagers. Cohort data provides a unique opportunity to temporally explore the impact of early adversity on later outcomes. Such data also allows us to explore whether particular risk profiles in early childhood might be associated with particular outcomes (e.g., mental health and particular risk behaviors; Dunn et al., 2011), providing important information for informing the specificity of targeted intervention.

## Method

### Data

Data were from the UK's Millennium Cohort Study (MCS) of approximately 19,000 children born between September 2000 and January 2002 (Connelly & Platt, 2014; Plewis, 2007). Cohort members and their families were surveyed when children were aged 9 months (MCS Wave 1; MCS1, 18,552 families), and subsequently at ages 3, 5, 7, 11, and 14 years (MSC Waves 2–6; MSC2–MSC6). Children born in the United Kingdom between 2000 and 2002 were recruited. The study used stratified sampling by electoral ward, and disproportionately stratified to oversample children living in areas with high ethnic minority populations or in disadvantaged communities.

For each wave of the survey, the child's main caregiver (primarily mothers) and their partner (if applicable; primarily biological fathers) were interviewed and carried out self-completion questionnaires relating to sociodemographic, health, and psychosocial information. Child-report (cohort member) data was also collected in the later waves. The present study uses self-report main caregiver data from 9 months (MCS1; achieved sample 18,552 families), 3 years (MCS2; 15,590 families), and 5 years (MCS3; 15,246 families) and cohort-member reported data at 14 years (MCS6; 11,726 families; Connelly & Platt, 2014; Ipsos MORI, 2017; Plewis, 2007; University of London, Institute of Education, Centre for Longitudinal Studies, 2012a, 2012b).

### Participants

We looked at data at 9 months, 3 years, and 5 years to determine early disruptive environments that could have long-term impacts on the cohort child's development. In total, data from 18,793 families were included in the latent class analysis as they had valid data from at least one time point. Families were excluded prior to analysis if the main respondent was not consistent across the three time points. We did not consider changes in the main respondent to provide equivalent data. In total, 99.5% of main respondents were the biological mother. This left a sample size of 18,132 families at 9 months, 15,139 (83%) families at 3 years, and 14,821 (82%) families at 5 years.

There were a total of 10,900 (60%) adolescents who completed at least part of the self-report questionnaire battery at age 14. These were either single-born children or the first named target child in multiple-birth households. We did not assess twins and triplets together due to the computational difficulties of adjusting for relatedness in addition to using survey estimation techniques, which is recommended when analysing data from the MCS (Ketende & Jones, 2011). The age 14 sample was evenly split between the genders (male = 5,419, 49.7%; female = 5,481, 50.3%).

### Measures

#### Latent transition analysis indicators

We used six indicators of early life adversity that were consistently measured across all three time points (9 months, 3 years, and 5 years). An additional seventh indicator of parental behavior was included at each time point, but this differed from 9 months to age 3 and 5 years. We used maternal attachment at 9 months and strict parental discipline at age 3 and 5 years.

##### Homelessness

Homelessness was defined as having to move out of a residence without another permanent place to live. At 9 months, the time frame was defined from the birth of the cohort child. For age 3 and 5 years, this time frame was defined as since the last interview.

##### Poverty

Poverty was measured based on the Organisation for Economic Co-operation and Development's definition of the family earning 60% less than the median income at the time of the interview. The rates found at all time points were comparable to the overall UK trends of children in relative poverty in the 2000s (McGuinnesss, 2016).

##### Parent relationship status

Parental relationship status was obtained through a derived variable at each time point (compiled by the Centre for Longitudinal Studies), which indexed the parents/caregivers in the household and their biological relation to the cohort child. This was categorized as the household containing both biological parents, households with two caregivers (at most one a biological parent; at 5 years, 737 children had one biological parent and a step parent/partner while an additional 35 had two other parent figures, but no biological parent), and households with a single parent (at 5 years, 3,012 had one natural parent and 18 had another single-parent figure).

##### Parental relationship conflict

Four questions from the Golumbok Rust Inventory of Parental State (Rust, Bennun, Crowe, & Golombok, 1990) were used to measure negative aspects of the parental relationship (whether with biological parents or nonbiological parents). These items were measured on a 5-point scale (*strongly agree* to *strongly disagree*) and were summed to create a total score (α = 0.74 for 9 months, α = 0.81 for 3 years; α = 0.80 for 5 years). We determined the top 10% of the distribution to be “high conflict,” which is consistent with similar definitions in the previous literature (Slopen et al., 2014), and the remainder to be low/medium conflict. As single-parent households would not have filled in these questions without a life partner, a third category of “no partner” was also used. This led to a three-category variable: no partner (no parental conflict by default), low/medium conflict (or not high conflict), and high conflict.

##### Reported use of force by partner

This indicator was whether the partner of the main respondent had ever used force against him or her. This was defined as grabbling, pushing, shaking, hitting, or kicking.

##### Parent mental health

A consistent standardized measure of psychological distress or depressive or anxiety symptoms was not available across all three time points. We, therefore, used a follow-up question of whether the main respondent was currently being treated for depression or serious anxiety. This provided a comparable rate across the three time points (see Table 1), as well as being a stringent measure of current mental difficulties, with only high-threshold cases being flagged.

**Table 1.** Fit Indices for 9-month, 3-year, and 5-year latent class solutions

9 Month	AIC	BIC	BICSSA	Entropy	VLMH test	LMH adj test	PBLRT	Class size
1 class	96063.59	96133.84	96105.24	—	—	—		18132
2 class	75621.46	75769.76	75709.38	1	<0.001	<0.001		14965/3167
3 class	74855.68	75082.04	74989.88	0.783	<0.001	<0.001		3167/13419/1546
4 class	74790.37	75094.78	74970.84	0.807	0.0009	0.001	<0.001	13702/380/883/3167
5 class	74788.48	75170.95	75015.23	0.827	0.0016	0.0017	1	582/513/12752/3167/1118
6 class	74798.69	75259.21	75071.71	0.785	0.2449	0.2485	0.6	656/2511/513/1118/12752/582
**3 Years**								
1 class	82125.66	82194.29	82165.69	—	—	—		15139
2 class	65690.91	65835.79	65775.4	1	<0.001	<0.001		2744/12395
3 class	65048.87	65270	65177.84	0.759	<0.001	<0.001		1191/11209/2739
4 class	64931.92	65229.3	65105.36	0.771	<0.001	<0.001		11271/2738/844/286
5 class	64915.08	65288.7	65132.99	0.75	1	1	<0.001	2517/221/844/286/11271
**5 Years**								
1 class	87939.16	88007.59	87978.99	—	—	—		14821
2 class	70629.07	70773.54	70713.16	1	<0.001	<0.001		2963/11858
3 class	70012.76	70233.27	70141.11	0.73	<0.001	<0.001		2962/1378/10481
4 class	69821.3	70117.84	69993.91	0.796	<0.001	<0.001	<0.001	960/2963/10554/344
5 class	69813.69	70186.28	70030.56	0.756	0.0016	0.0017	1	428/10554/960/2535/344
6 class	69814.3	70262.92	70075.42	0.687	0.3052	0.3091		1566/2535/428/9641/333/318

*Note*: AIC, Akaike information criteria. BIC, Bayesian information criteria. BICSSA, Bayesian information criteria sample size adjusted. VLMH, Vuong–Lo–Mendel–Rubin likelihood ratio test. LMH adj Test, Lo–Mendell–Rubin adjusted likelihood ratio test. PBLRT, parametric bootstrapping likelihood ratio test.

##### Maternal attachment

The Condon Maternal Attachment Questionnaire was used at 9 months to provide a measurement of attachment (Condon & Corkindale, 1998). There were 6 items, which were summed to create a composite score (range: 6–23), where higher scores indicated better attachment. The reliability was quite low (α = 0.51) but this is common for scales with fewer than 10 items (Pallant, 2005). As an alternative for low item scales, the average interitem correlations were consulted and found to be just below the recommended range (.20–.40) at .18 (Briggs & Cheek, 1986). We took the lowest 10% of the sample on this questionnaire as a measurement of “poor attachment,” operationally defined as less than 16.

##### Harsh parental discipline

We used three indicators from the Straus Parental Discipline and Conflicts scale (Straus & Hamby, 1997) to create a measurement of particularly harsh parental discipline, in line with previous research on this sample (Flouri & Midouhas, 2017). These items were summed to create a composite (range: 3–15) with higher scores indicating harsher parental discipline. The reliability was lower than acceptable (α = 0.65 at age 3; α = 0.66 at age 5), but the average interitem correlations were within the recommended range (.38 at age 3 and 5; Briggs & Cheek, 1986). In line with all other indicators, we created a binary variable from this measurement with the top 10% of families being characterized by “harsh parental discipline.” This was defined as above 12 at age 3 and above 10 at age 5. The cutoffs varied as the top 10% cutoff differed at each age.

#### Risk-taking behavior outcome indicators

At age 14, adolescent self-report interview data was utilized to measure a range of developmentally maladaptive outcomes, including risk behaviors, such as early sexualization and substance use. All ratings were yes/no dichotomy responses unless otherwise indicated.

##### Early sexualization

This variable measured any report of either oral sex (performed on someone else or had performed on them) or sexual intercourse with another young person.

##### Substance use

For alcohol consumption, we looked only at binge drinking, which was defined as having ever had five or more alcoholic drinks at a time, with an alcoholic drink defined as a half a pint of lager, beer, or cider; one alcopop; a small glass of wine; or a measure of spirits. For illegal drugs, a specific question on cannabis use was combined with a question for “any other illegal drug (such as ecstasy, cocaine, and speed)?” This created a composite of any illegal drug use combining cannabis and other illegal drugs. All of these questions were in relation to any use across their lifetime.

#### Antisocial and criminal behavior outcome indicators

Antisocial and criminal behavior outcomes included antisocial behavior, which is likely a criminal act (property damage or shoplifting) and criminal activity (which differed from antisocial behavior in the severity of the behavior) and contact with the police. All ratings were yes/no dichotomy responses unless otherwise indicated.

##### Antisocial behavior

Cohort member reported vandalizing or damaging property, shoplifting, and hacking in the past 12 months. For property damage, we combined the rating of vandalizing (“Have you written things or spray painted on a building, fence, or train or anywhere else where you shouldn't have?”) with damaging property (“Have you on purpose damaged anything in a public place that didn't below to you, for example by burning, smashing, or breaking things like cars, bus shelters, and rubbish bins?”) into one variable. Shoplifting was measured by a question about taking something from a shop without paying for it. Hacking was measured by a question asking whether the cohort member “had accessed, or hacked into, someone else's computer, e-mail, or social networking account without their permission.”

##### Criminal activity

This included whether the cohort member carried a weapon, assaulted or used a weapon against someone, had stolen property, and gang membership. The question relating to carrying a weapon related to whether the cohort member ever “carried a knife or other weapon for your own protection, because someone else asked you to, or in case you get into a fight?” We combined into one variable responses about the cohort member having “pushed or shoved/hit/slapped/punched someone” or “used or hit someone with a weapon” in the past 12 months. Whether the cohort member had stolen property was defined as “stolen something from someone, e.g., a mobile phone, money” in the past 12 months. Gang membership combined current gang membership with past gang membership. Gangs were defined as “groups of young people who hang around together, and have a specific area or territory; have a name, a color, or something else to identify the group; possibly have rules or a leader; who may commit crimes together.”

##### Contact with the police

Police contact was defined as being questioned by the police, given warning by the police, or being arrested. The cohort members was asked if they were ever “stopped and questioned by the police” and whether they had “ever been given a formal warning or caution by a police officer.” For the arrest, cohort members were asked if they had “ever been arrested by a police officer and taken to a police station.”

#### Criminal victimization outcome indicators

This included whether the cohort members had been assaulted or had a weapon used against them, whether they had had something personal stolen from them (e.g., mobile phone or money), and whether someone had “made an unwelcome sexual approach to you or assaulted you sexually.” We combined the positive ratings for assault (“Has anyone been physically violent towards you, e.g., pushed, shoved, hit, slapped, or punched you?”) and assault with a weapon (“Has anyone hit you with or used a weapon against you?”) into a single measure.

#### Mental health outcome indicators

Self-reported mental health outcomes at 14 were depressive symptoms, self-esteem, and self-harm.

##### Self-harm

Measured by a positive response to the following question: “In the past year have you hurt yourself on purpose in any way?”

##### Depressive symptoms

A 13-item version of the Moods and Feelings Questionnaire was administered to the cohort members (Costello & Angold, 1988). Respondents indicate how they had been feeling or acting in the past 2 weeks across the 13 items measuring depressive symptoms on a 3-point scale (*not true*, *sometimes*, or *true*). Total (summed) score was calculated if the cohort member had completed all questions (α = 0.93).

##### Self-esteem

Five items from the Rosenberg Self-Esteem Scale (Rosenberg, 1965) were used, rated on a 4-point scale (*strongly agree*, *agree*, *disagree*, or *strongly disagree*). Total (summed) score was calculated if the cohort member had completed all questions (α = 0.91). Recent research highlights the importance of measuring mental wellness/low mental health risk alongside negative emotional symptoms in adolescence (St Clair et al., 2017).

### Data analysis

#### Latent class analysis

The latent class analyses were conducted in Mplus version 8 (Muthén & Muthén, 1998–2017). Latent class analysis at each time point was preferred over an overall latent class analysis as we wished to evaluate dynamic change across the three age points. Combining indicators across all of early childhood to produce a single latent class structure would blur the specific risks at a single time point and the dynamic and changing nature of the risk across development. Six of the seven latent class indicators at 9 months, age 3 years, and age 5 years were equivalent in terms of their wording and measured the same early life adversity indicators at each time point. The seventh indicator (suboptimal parenting) differed at 9 months (poor maternal attachment), but was equivalent in wording between 3 and 5 years (harsh parental discipline). All indicators were categorical and were thus set to be categorical within MPlus. Three latent class analyses were run for the 9-month, 3-year, and 5-year data, respectively. The estimator was maximum likelihood with robust standard errors. The number of random starts in the initial stage was 200, with 50 optimizations in the final stage.

See Table 1 for the fit indices for each latent class analysis. The fit indices evaluated were the Bayesian information criterion (BIC), sample-size adjusted BIC, and the Akaike information criterion, where lower values indicated a more parsimonious model. We also evaluated the Vuong–Lo–Mendell–Rubin and Lo–Mendell–Rubin adjusted likelihood ratio tests (LRT), which evaluated whether an additional class significantly improved the model fit. Finally, to determine the final class solution accepted between two alternatives, we looked at specific model comparisons using the more computational intensive parametric bootstrapping LRT procedure, which have been shown to be the most sensitive and valid LRT (Dziak, Lanza, & Tan, 2014). Each time point indicated either a four- or five-class solution was optimal. The four-class solution had lower BIC fit indices than the five-class solution at all time points, indicating the four-class solution was a more parsimonious fit to the data. The Akaike information criterion, however, indicated the five-class solution was more parsimonious than the four class. There was again conflicting information for the four- and five-class solutions with the LRTs. On balance, however, the four-class solution was chosen, as both the BIC and the parametric bootstrap LRT are considered to be more sensitive methods of evaluating model comparison. The four-class solution had the highest entropy at each time point as well, indicating the best differentiation between the classes. The entropy levels were near or at .80, indicating good differentiation between the classes. In addition, the four-class solution also produced equivalent classes across all three time points.

The results of the final latent class analyses were saved using the SAVEDATA command. This output was then imported back to Stata and merged with the database including the indicator and outcome variables.

#### Statistical analysis

All subsequent analysis was conducted in StataMP 15.1 (StataCorp, 2017). As suggested by the MCS documentation, survey weights were used for all subsequent analyses and mean/proportion estimations (Ketende & Jones, 2011). The weights defined in the svyset command adjust for attrition based on the latest data wave used in the analysis. For example, weights for the 9-month data when analyzing the 9-month indicators. For all outcome variables, weights for the 14-year data were used. The svyset command also adjusts the estimates with a population correction factor to produce UK population-level estimates. We evaluated whether each class had either decreased or increased levels of the indicator variables when compared to the remainder of the sample (e.g., all other classes combined). A dummy variable for each class at each time point was created comparing that specific class to all the other classes combined. The outcome variables were then compared to different combinations of the class variation of stability across development detailed in the results. All outcome variables were evaluated individually across the subgroup combinations using the mlogit command. A specific class was chosen to be the comparison class, as specified in the results. The relative-risk ratio was reported for each specific subgroup contrast with the comparison class. This represents the probability of the outcome event occurring in the group of interest when compared to the comparison group. Thus, a relative-risk ratio of 2 indicates that the outcome was twice as likely to occur in the class of interest than in the specified comparison class.

The gender of the adolescent cohort member was controlled in all analyses involving outcome variables. In addition, a Gender × Outcome Variable interaction was investigated and mentioned in the results only when significant. If significant, this indicated that the gender distribution experiencing the outcome differed in a specific class in comparison to the reference class. The significant interactions are explored further in the Results section. Occasionally, significant interactions were driven by no endorsement in one group, when resulted in a significant decrease even when endorsement in the comparison group was only 1% or lower. In these cases, we do not report the interaction in the results (as we view this as a limit of the sample size rather than a real effect) but explain the interaction in the online-only Supplementary Materials. When evaluating transitions between classes, we separated the effect of transition by age 3 (so between the 9-months and 3-year time waves) and the transition by age 5 (so between the age 3- and 5-year time waves).

Due to the amount of comparisons within the results, we have reduced our significance level to .01. However, as many readers may be interested in *p* values below the traditional value of .05, the statistics for these results are provided, and these results are treated as marginal. We do not discuss these marginal results in the written text, but they can be evaluated in all tables. No statistics are provided for results with *p* values above .05. Only gender interactions considered significant (at .01 levels) are reported.

## Results

See Table 2 and Table 3 for the overall rates of the early adversity indicators as well as the age 14 outcome variables. All findings related to latent class indicators in a specific latent class were in comparison to the combined remaining latent classes.

**Table 2.** Latent classes at 9 months (T1), 3 years (T2), and 5 (T3) years by latent class indicators

	9 Months					3 Years					5 Years				
	Overall results	Two-parent/ econ. adv. (*n* = 13,702; 75.6%)	Two-caregiver/econ. hardship (*n* = 380; 2.10%)	Two-parent/ high-conflict (*n* = 883; 4.9%)	Single-parent/ econ. hardship (*n* = 3,167; 17.5%)	Overall results	Two-parent/ econ. adv. (*n* = 11,271; 74.5%)	Two-caregiver/ econ. hardship (*n* = 286; 1.9%)	Two-parent/ high-conflict (*n* = 844; 5.6%)	Single-parent/ econ. hardship (*n* = 2,738; 18.1%)	Overall results	Two-parent/ econ. adv. (*n* = 10,554; 71.2%)	Two-caregiver/ econ. hardship (*n* = 344; 2.3%)	Two-parent/ high-conflict (*n* = 960; 6.5%)	Single-parent/ econ. hardship (*n* = 2,963; 20.0%)
Use of force	2.9%	1.8%***	0.7%^+^	33.2%***	0%	3.2%	2.0%***	0%	33.5%***	0.1%***	3.1%	1.7%***	0%	31.7%***	0%
Homelessness	0.92%	0.17%***	15.2%***	0.13%^+^	3.4%***	2.1%	0%	43.2%***	5.0%***	5.4%***	1.1%	0.2%***	9.9%***	0.7%	3.6%***
Depression	8.5%	5.8%***	4.5%^+^	43.8%***	12.3%***	8.1%	4.3%***	13.2%**	39.1%***	13.9%***	8.0%	4.1%***	19.8%***	29.6%***	13.3%***
OECD poverty	30.0%	18.0%***	100%	39.8%***	82.8%***	29.5%	15.7%***	72.1%***	62.3%***	74.4%***	30.4%	15.0%***	94.2%***	51.3%***	71.0%***
Low attachment/ harsh parental discipline	10.4%	8.6%***	2.5%***	45.9%***	9.8%	7.5%	6.8%***	7.0%	14.1%***	8.8%	12.9%	10.7%***	14.5%	31.9%***	14.1%
Parent status															
Two natural parents	85.0%	99.8%	88.1%	99.9%	0%	79.2%	98.6%	31.8%	93.1%	0%	74.7%	96.0%	4.3%	95.7%	0%
Two parent figures	0.38%	0.2%	11.9%	0.07%	0%	2.7%	1.4%	67.5%	6.7%	0%	5.4%	3.9%	95.7%	4.2%	0%
One parent figure	14.6%	0%	0%	0%	100%	18.1%	0%	0.7%	0%	100%	19.8%	0.02%	0%	0%	100%
Parent relationship															
No Partner	—	0%	0%	0%	100%	—	0%	0%	0%	100%	—	0%	0%	0%	100%^±^
Not high conflict	86.5%	93.3%	15.5%	5.5%	0%	84.4%	90.2%	90.6%	4.8%	0%	84.7%	91.9%	83.4%	7.5%	0%^±^
High conflict	13.5%	6.7%***	84.5%***	94.5%***	0%	15.6%	9.8%***	9.4%^+^	95.1%***	0%	15.3%	8.1%***	16.6%	92.4%***	0%

*Note*: ^±^These figures were rounded up to 100%; there were a small number of individuals in the other class, but not sufficient numbers to equal over 0.1%. *p < .05. **p < .01. ***p < .001.

**Table 3.** Transition between 9-month and 3-year latent classes and between 3-year and 5-year latent classes

	3 Years				
9 Months	Two-parent/econ. adv.	Two-caregiver/econ. hardship	Two-parent/high-conflict	Single-parent/econ. hardship	Total
Two-parent/econ. adv.	9870 (87.7%)	122 (1.1%)	510 (4.5%)	756 (6.7%)	11,258
Two-caregiver/econ. hardship	157 (56.27%)	18 (6.45%)	45 (6.1%)	59 (21.2%)	279
Two-parent/high-conflict	414 (59.1%)	15 (2.1%)	155 (22.1%)	117 (16.7%)	701
Single-parent/econ. hardship	476 (21.3%)	91 (4.1%)	92 (4.1%)	1581 (70.6%)	2,240

	5 Years				
3 Years	Two-parent/econ. adv.	Two-caregiver/econ. hardship	Two-parent/high-conflict	Single-parent/econ. hardship	Total
Two-parent/econ. adv.	8951 (88.6%)	65 (0.6%)	583 (5.8%)	507 (5.0%)	10,106
Two-caregiver/econ. hardship	111 (46.4%)	79 (33.1%)	10 (4.2%)	39 (16.3%)	239
Two-parent/high-conflict	357 (47.7%)	23 (3.1%)	234 (31.2%)	135 (18.0%)	749
Single-parent/econ. hardship	345 (15.1%)	120 (5.3%)	38 (1.7%)	1780 (78.0%)	2,283

### Latent class analysis

Details of the indicator variables for all four classes at 9 months, 3 years, and 5 years are given in Table 2. The classes were broadly comparable across the three time points. The first class was characterized by a two-parent household (overwhelmingly two biological parents) with reduced rates of risk factors (i.e., low adversity). Two additional classes were characterized by a two-parent household, but with other elevated adversity risk factors that encompassed two distinct trajectories (one characterized by economic hardship and the other by high parental conflict). The fourth class was characterized as a single-parent household with elevated risk factors (i.e., particularly high adversity).

Class 1 was the largest class and was categorized by two parent figures, typically both biological parents, with reduced risks for most other risk factors, including reduced rates of poverty, homelessness, and parental treatment for depression. There were also low rates of both use of force and high conflict within the parental relationship. The rates of poor attachment and use of harsh discipline at age 3 was also reduced, although the use of harsh discipline was not significantly reduced at age 5. This low-adversity class is labeled the *two-parent/economic advantage* class. This class reduced in numbers across the three time points, from 75.6% of the sample at 9 months to 71.2% of the sample at 5 years.

Class 2 was the smallest class, ranging from 1.9% (9 months) to 2.3% (5 years) of the sample. This class was characterized by a two-parent household, which increasingly encompassed stepparents rather than two biological parents, and consistent indicators of economic disadvantage. This class is labeled the *two-caregiver/economic hardship* class. There were consistent elevated rates of poverty and homelessness in this class (e.g., 70%–100% living in poverty across the three time points). Overall, while poverty was high, there were low levels of use of force within the parental relationship and also reduced rates of poor attachment at 9 months. There were some differences in the patterns between the 9- month class and age 3 and 5 classes. There were reduced rates of treatment for depression at 9 months, whereas there were increased rates at 3 and 5 years. Conversely, while there was a very high rate of parental conflict (without force) within the parental relationship at 9 months, overall there were low rates of parental conflict at 3 years and 5 years, potentially reflecting the breakdown of the original parental relationship (with increasing proportions of stepparents at the later time points). There was no difference found in the rate of harsh parental discipline at 3 and 5 years and reduced prevalence of poor attachment at 9 months.

Class 3 ranged from 4.9% of the sample at 9 months to 6.5% of the sample at 5 years and was characterized by generally increased risk factors. Parents in this class were likely to be the biological parents of the child, while the class was particularly characterized by high levels of use of force and high conflict within the parental relationship. There was also increased rates of poverty and very high rates of treatment for depression. Parents in this class were much more likely to experience poor attachment and use harsh parental discipline with their children. This class was labeled as the *two-parent/high-conflict* class.

Class 4 was the *single-parent/economic hardship* class and consisted of only one resident parent, usually a biological mother. The prevalence of this class was relatively stable, but increased slightly from 17.5% of the sample at 9 months to 20% at 5 years. By default, there was very little evidence of use of force by a partner nor any relationship conflict in this class. However, this class was also defined by increased rates of homelessness and poverty, indexing economic disadvantage. These parents were also more likely to be in current treatment for depression or severe anxiety. There were neither reductions nor increases in the rate of poor attachment and harsh parental discipline in this group.

#### Transitions between classes

See Table 3 for the transition between classes and online only Supplemental Table S.1 for numbers for each transition pattern across all three time points. The majority of the sample (*n* = 9,208; 71.7%) remained in the same class across the three time points (9 months, 3 years, and 5 years), mainly within the low-adversity *two-parent/economic advantage* or high-adversity *single-parent/economic hardship* classes. Thus, approximately one-third of the sample showed movement into another class by either 3 years of age or 5 years of age. Of those in a two-parent household at 9 months 7.6% (*n* = 932) transitioned to a single-parent household at 3 years, while 6.1% (*n* = 681) transitioned to a single-parent household by 5 years. These transitions were mostly from the higher risk two-parent groups (i.e., where there was economic disadvantage or high conflict), rather than the lower risk two-parent group. There was greater movement of families resolving situations of conflict and moving into a low-risk grouping, than families transitioning from a low-risk status to a higher risk environment. Similarly, single parents were more likely to either remain in a single-parent household or move into a *two-parent/economic advantage* environment, with low rates of transition into a two-parent higher risk environment (see Table 3).

#### Stable class membership and adolescent outcomes

Prior to investigating how transitioning between the latent classes influences adolescent outcomes, we first investigated how the individuals who remained stable in the class membership differed on these outcomes. Only the largest two classes were sufficiently large to evaluate individuals who remained in that class throughout all three time points. Therefore, only the stable high-adversity *single-parent/economic hardship* class was compared to the stable low-adversity *two-parent/economic advantage* class. As the labels suggest, these classes differ in composition (two parent vs. single parent) and economic hardship (low rates of poverty vs. higher rates of poverty; see Table 2). See Table 4 for a summary of the findings.

**Table 4.** Risk behavior, criminal behavior, victimization, and mental health outcomes for the entire sample and stable “two-parent/economic advantage” and stable “single-parent/economic hardship” latent classes (n = families with data at age 14)

	Overall	Stable two- parent/econ adv. (*n* = 6,084)	Stable single- parent/econ hardship (*n* = 638)	Comparison (RRR/ coefficient and CI)

Risk behavior outcomes				
*Early sexualization*				
Oral sex/intercourse^±^	3.4%	2.6%	4.3%	1.70 [1.03, 2.81]^+^
*Substance use*				
Ever binge drinking	10.8%	8.6%	11.8%	*ns*
Cannabis/illegal drug use^±±^	5.7%	3.5%	9.3%	2.84 [1.92, 4.20]***
Antisocial and criminal behavior outcomes				
*Antisocial behavior*				
Cohort member (CM) vandalized/damaged property^±±±^	5.4%	3.6%	8.7%	2.54 [1.66, 3.89]***
CM shoplifted	3.7%	3.0%	6.0%	2.08 [1.39, 3.12]***
Hacking	4.7%	4.1%	6.2%	*ns*
*Criminal activity*				
CM had carried weapon	2.7%	1.7%	4.6%	2.76 [1.67, 4.57]***
CM has hit/assaulted or used weapon against someone^±±±^	31.9%	28.2%	39.4%	1.72 [1.38, 2.14]***
CM has stolen property	1.2%	1.0%	2.4%	2.53 [1.26, 5.11]**
CM is or was in gang	3.9%	2.1%	7.9%	3.88 [2.42, 6.23]***
*Contact with police*				
CM questioned by police	16.6%	11.1%	23.2%	2.45 [1.91, 3.14]***
CM given warning by police	9.3%	5.4%	15.4%	3.22 [2.32, 4.46]***
CM arrested by police	1.4%	0.6%	1.8%	2.96 [1.29, 6.79]^+^
Criminal victimization outcomes				
CM was violently assaulted/had weapon used on^∝^	23.8%	21.8%	26.7%	1.33 [1.05, 1.68]^+^
CM had something stolen	7.8%	6.6%	8.6%	*ns*
CM was sexually assaulted	2.8%	2.5%	3.6%	*ns*
Mental health outcomes				
Past-year self-harm	15.4%	13.0%	18.5%	1.54 [1.16, 2.02]**
MFQ depression symptoms	5.71 (.08)	5.22 (.10)	6.29 (.37)	.03 [.01, .05]**
Rosenberg self-esteem	10.49 (.05)	10.71 (.06)	10.0 (.16)	–.09 [–.13, –.05]***

*Note*: ^±^Oral sex: 3.1%; intercourse: 2.1%. ^±±^Cannabis use: 5.6%; illegal drug use: 0.8%. ^±±±^CM vandalized: 2.9%; CM damaged property: 3.7%. ^±±±±^CM has hit or assaulted someone: 31.8%; CM has used weapon against someone: 1.1%. ∝CM was violently assaulted: 23.3%; CM was hit or had a weapon used: 3.6%. ^+^*p* < .05. **p < .01. ***p < .001.

As there were only 7 individuals who were consistently in the *two-caregiver/economic hardship* latent class and 76 who remained stable in the *two-parent/high-conflict* latent class, we could not consider these as stable classifications (i.e., there was insufficient stability to evaluate a “stable” *two-caregiver/economic hardship* class and a *two-parent/high-conflict* class across all three time points). We instead investigated the effect of these classes at both 3 and 5 years of age, in comparison to the comparable *two-parent/economic advantage* class at each age. These allowed us to broadly explore the impact of being in a two-parent home with and without adversity. We do not report the results from 9 months, as these were broadly replicated at 3 years. See Table 5 for a summary of the results.

**Table 5.** Risk behavior, criminal behavior, victimization, and mental health outcomes for the entire sample, “two-parent/economic advantage,” “two-caregiver/economic hardship,” and “two-parent/high-conflict” classes at ages 3 and 5 (n = families with data at age 14)

	Overall	Two-parent/ econ. adv. age 3 (*n* = 7,820)	Two-caregiver/econ. hardship age 3 (*n* = 159)		Two-parent/high-conflict age 3 (*n* = 534)		Two-parent/ econ. adv. age 5 (*n* = 7,660)	Two-caregiver/econ. hardship age 5 (*n* = 203)		Two-parent/high-conflict age 5 (*n* = 662)	
Risk behavior outcomes	%	%	%	RRR/Coef. & CI	%	RRR/Coef. & CI	%	%	RRR/Coef. & CI	%	RRR/Coef. & CI
*Early sexualization*											
Oral sex/intercourse	3.4%	2.9%	6.1%	*ns*	4.8%	1.71 [1.07, 2.74]^+^	3.0%	5.5%	*ns*	4.4%	*ns*
*Substance use*											
Ever binge drinking	10.8%	9.6%	11.1%	*ns*	13.1%	1.42 [1.04, 1.9]^+^	9.1%	15.8%	1.87 [1.17, 3.0]**	14.5%	1.69 [1.19, 2.39]**
Cannabis/illegal drug use	5.7%	4.1%	6.2%	*ns*	8.5%	2.15 [1.45, 3.19]***	4.2%	9.1%	2.28 [1.27, 4.08]**	8.6%	2.14 [1.35, 3.42]**
Antisocial and criminal behavior outcomes											
*Antisocial behavior*											
Cohort member (CM) vandalized/damaged property	5.4%	4.4%	7.1%	*ns*	6.8%	1.58 [1.07, 2.34]^+^	4.1%	8.0%	1.99 [1.07, 3.69]^+^	8.8%	2.26 [1.44, 3.53]***
CM shoplifted	3.7%	3.1%	0.6%	0.19 [.04, 84]^+^	2.9%	*ns*	3.0%	3.8%	*ns*	3.6%	*ns*
Hacking	4.7%	4.4%	1.4%	*ns*	4.4%	*ns*	4.3%	3.0%	*ns*	6.8%	1.65 [1.12, 2.43]^+^
*Criminal activity*											
CM had carried weapon	2.7%	1.8%	4.1%	*ns*	6.2%	3.60 [2.07, 6.27]***	2.2%	8.0%	3.73 [1.98, 7.39]^+^	2.5%	*ns*
CM has hit/assaulted or used weapon against someone	31.9%	29.8%	30.4%	*ns*	33.6%	*ns*	29.7%	42.0%	1.67 [1.15, 2.45]**	35.8%	1.36 [1.10, 1.69]**
CM has stolen property	1.2%	1.1%	2.6%	*ns*	1.2%	*ns*	1.1%	2.6%	*ns*	1.3%	*ns*
CM is or was in gang	3.9%	2.7%	4.3%	*ns*	7.2%	2.83 [1.79, 4.47]***	2.5%	8.2%	3.49 [1.87, 6.50]***	6.0%	2.45 [1.38, 4.38]**
*Contact with police*											
CM questioned by police	16.6%	12.7%	26.6%	2.40 [1.53, 3.77]***	22.7%	2.04 [1.54, 2.69]***	12.7%	26.3%	2.40 [1.63, 3.55]***	19.7%	1.71 [1.29, 2.26]***
CM given warning by police	9.3%	6.8%	15.7%	2.45 [1.38, 4.35]**	13.6%	2.17 [1.49, 3.14]***	6.6%	17.6%	2.97 [1.77, 4.99]***	12.5%	2.04 [1.48, 2.81]***
CM arrested by police	1.4%	0.8%	3.0%	3.56 [1.01, 12.50]^+^	2.8%	3.54 [1.27, 9.84]^+^	0.9%	1.4%	*ns*	2.3%	2.62 [1.24, 5.55]^+^
Criminal victimization outcomes											
CM was violently assaulted/had weapon used on	23.8%	22.3%	27.1%	*ns*	27.3%	1.32 [1.04, 1,69]^+^	22.6%	34.4%	1.76 [1.20, 2.58]**	22.4%	*ns*
CM had something stolen	7.8%	7.1%	9.3%	*ns*	9.0%	*ns*	7.5%	12.5%	1.86 [1.04, 3.31]^+^	7.8%	*ns*
CM was sexually assaulted	2.8%	2.6%	1.2%	*ns*	5.0%	2.0 [1.19, 3.37]**	2.5%	3.3%	*ns*	3.4%	*ns*
Mental health outcomes											
Past-year self-harm	15.4%	13.6%	16.6%	*ns*	18.8%	1.48 [1.14, 1.93]**	13.7%	26.4%	2.49 [1.62, 3.81]***	18.4%	1.42 [1.10, 1.85]**
MFQ depression symptoms	5.71 (.08)	5.41 (.09)	6.13 (.59)	*ns*	6.81 (.34)	.04 [.02, .06]^+^	5.37 (.09)	6.89 (.61)	.05 [.02, .08]**	6.06 (.31)	.02 [.003, .03]^+^
Rosenberg self-esteem	10.49 (.05)	10.65 (.05)	10.29 (.29)	*ns*	10.06 (.15)	–.07 [–.11, –.04]***	10.68 (.05)	9.80 (.25)	–.12 ] [–.17, –.07]***	10.31 (.14)	.–05 [–.08, –.01] ^+^

^+^*p* < .05. **p < .01. ***p < .001.

#### “Single-parent/economic hardship” class versus “two-parent/economic advantage” class

##### Risk behavior outcomes

With regard to engaging in potentially risky behaviors, adolescents in the stable *single-parent/economic hardship* class were significantly more likely to engage in illegal drug use compared to those in the *two-parent/economic advantage* class.

##### Antisocial and criminal behavior outcomes

Adolescents in the stable *single-parent/economic hardship* class were significantly more likely to have vandalized or damaged property and shoplifted when compared to the stable *two-parent/economic advantage* class. They were also significantly more likely to have carried a weapon, hit or assaulted someone, have stolen property from someone, and be in a gang.

Adolescents within the stable *single-parent/economic hardship* class were significantly more likely to be questioned, cautioned, or given a warning by the police (as compared to the *two-parent/economic advantage* class).

##### Criminal victimization outcomes

There were no significantly increased criminal victimization outcomes for adolescents in the stable *single-parent/economic hardship* class when compared to those in the *two-parent/economic advantage* class.

##### Mental health outcomes

Those in the stable *single-parent/economic hardship* class also had significantly elevated rates of self-harm, increased depressive symptoms, and reduced self-esteem than adolescents in the stable *two-parent/economic advantage* class.

#### “Two-caregiver/economic hardship class” versus “two-parent/economic advantage” class

##### Risk behavior outcomes

There were no increases in early sexual behavior associated with being in the *two-caregiver/economic hardship* class versus the *two-parent/economic advantage* (i.e., low adversity) class at 3 years of age, but those who were in the economic hardship class at 5 years had significantly increased rates of binge drinking and illegal drug use in adolescence.

##### Antisocial and criminal behavior outcomes

Adolescents who were in the *two-caregiver/economic hardship* class at age 3 had a significantly increased risk of being questioned and warned by the police when compared to the *two-parent/economic advantage* class. There was a significantly increased risk of assaulting someone, being in a gang, and being questioned and warned by the police in this class at age 5 (when compared to the *two-parent/economic advantage* class).

##### Criminal victimization outcomes

There were no significant differences associated with the *two-caregiver/economic hardship* class versus the *two-parent/economic advantage* class at age 3, but there were significantly increased rates of being assaulted, associated with being in the economic disadvantage class at age 5 when compared to the economically advantaged class at this age point.

##### Mental health outcomes

There were no significant differences in mental health outcomes associated with the *two-caregiver/economic hardship* class at age 3 (as compared to the *two-parent/economic advantage* class), but there was an increase in self-harm, depressive symptoms, and reduced self-esteem in the *two-caregiver/economic hardship* class at age 5 (compared to the *two-parent/economic advantage* class).

#### “Two-parent/high-conflict class” versus “two-parent/economic advantage” class

##### Risk behavior outcomes

There were significantly increased rates of binge drinking (marginal at age 3) and illegal drug use at both ages when compared with the equivalent low-adversity class.

##### Antisocial and criminal behavior outcomes

Adolescents who were in the *two-parent/high-conflict* class at age 3 were significantly more likely to carry a weapon, be involved in gangs, and be questioned and warned by the police when compared to the *two-parent/economic advantage* class. There was increased risk of vandalizing or damaging property, assaulting someone, being in a gang, and being questioned, and warned by the police in the *two-parent/high-conflict* class at age 5 when compared to the *two-parent/economic advantage* class.

We found a gender interaction with the *two-parent/high-conflict* class at age 5 and assaulting someone, *B* = 0.58, 95% confidence interval (CI) [0.15, 1.02], *p* < .01. While there was no increased rate of assaulting people in boys in the *two-parent/high-conflict* class at age 5 in comparison to the *two-parent/economic advantage* class (42.1% vs. 40.6%, *p* = .67), there was an increased rate in girls in the *two-parent/high-conflict* class at age 5, 29.5% versus 18.0%, relative-risk ratio = 1.90, 95% CI [1.39, 2.61], *p* < .001.

##### Criminal victimization outcomes

There were no significant differences associated with the *two-parent/high-conflict* class at age 5, but there was an increased risk of being sexually assaulted associated with the *two-parent/high-conflict* class at age 3 when compared to the *two-parent/economic advantage* class at this age.

There was a significant gender interaction between being the victim of sexual assault and having been in the *two-parent/high-conflict* class at age 3, *B* = 1.89, 95% CI [0.48, 3.30], *p* < .01. This was due to equivalent (and low) rates of sexual assault in high-risk boys compared to low-risk boys at age 3 (0.05% vs. 1.3%), *p* = .15, whereas there was an increased rate in sexual assault in girls who were in the higher risk class at age 3 (10% vs. 4% in age 3 low-risk girls), relative-risk ratio = 2.59, 95% CI [1.47, 4.56], *p* < .005.

##### Mental health outcomes

There was a significantly increase in self-harm and reduced self-esteem (marginal at age 5) in the *two-parent/high-conflict* class at both ages when compared to the *two-parent/economic advantage* class.

#### Transitions between classes: Transition out of “two-parent/economic advantage” (low-adversity) class

We next looked at adolescents whose families were in the two-parent/economic advantage class at 9 months, but transitioned to either the *two-parent/high-conflict* class or the *single-parent/economic hardship* class either before age 3 or between age 3 and age 5 (i.e., transitioned from a lower to a higher risk class). There were insufficient numbers to evaluate transition into the *two-caregiver/economic hardship* class at either time point.

Approximately 6.7% of the low-adversity *two-parent/economic advantage* families at 9 months experienced a relationship breakdown and transitioned to a single-parent household before the child's 3-year assessment. This transition also saw them move in to a group categorized by high rates of economic disadvantage and increased parental mental health difficulties. Approximately 4.5% of the low-adversity *two-parent/economic advantage* families transitioned into a *two-parent/high-conflict* environment by age 3. This move represents a group who were largely still living with their partner (i.e., two biological parents), but there was substantial increase in parental conflict and economic hardship. A further 5.0% transitioned from the *two-parent/economic advantage* class to the *single-parent/economic hardship* class between the age 3 and 5 assessments. Finally, between 3 and 5 years old, 5.8% transitioned from the low-adversity *two-parent/economic advantage* class to the *two-parent/high-conflict* class. For these analyses the comparison group was young people who stayed stable in the lower risk *two-parent/economic advantage* group across all time points. See Table 6 for a summary of these results.

**Table 6.** Risk behavior, criminal behavior, victimization, and mental health outcomes for the entire sample, consistent low-risk “two-parent/economic advantage” families and movement away from consistent low-risk “two-parent/economic advantage” families (n = families with data at age 14)

	Overall	Consistent low-risk “two-parent/econ. adv.” (*n* = 6,084)	Low-risk → “two-parent/high-conflict” at age 3 (*n* = 330)		Low-risk → “single-parent/econ. hardship” by age 3 (*n* = 438)		Low-risk → “two-parent/high-conflict” at age 5 (*n* = 326)		Low-risk → “single-parent/econ. hardship” by age 5 (*n* = 261)	
Risk behavior outcomes	%	%	%	RRR/Coef. & CI	%	RRR/Coef. & CI	%	RRR/Coef. & CI	%	RRR/Coef. & CI
*Early sexualization*										
Oral sex/intercourse	3.4%	2.6%	3.3%	*ns*	3.8%	*ns*	6.0%	*ns*	2.2%	*ns*
*Substance use*										
Ever binge drinking	10.8%	8.6%	10.7%	*ns*	15.4%	1.95 [1.37, 2.76]***	14.8%	1.84 [1.10, 3.11]^+^	12.0%	*ns*
Cannabis/illegal drug use	5.7%	3.5%	5.7%	*ns*	7.8%	2.32 [1.47, 3.65]***	7.2%	*ns*	2.9%	*ns*
Antisocial and criminal behavior outcomes										
*Antisocial behavior*										
Cohort member (CM) vandalized/damaged property	5.4%	3.6%	6.5%	1.83 [1.12, 2.99]^+^	6.6%	1.88 [1.15, 3.07]^+^	9.7%	2.85 [1.48, 5.46]**	5.0%	*ns*
CM shoplifted	3.7%	3.0%	3.1%	*ns*	5.6%^+^	1.88 [1.10, 3.22]^+^	4.0%	*ns*	2.2%	*ns*
Hacking	4.7%	4.1%	4.5%	*ns*	6.0%	*ns*	7.5%	1.88 [1.13, 3.14]^+^	4.0%	*ns*
*Criminal activity*										
CM had carried weapon	2.7%	1.7%	6.6%	3.99 [2.00, 7.97]***	4.3%	2.50 [1.27, 4.90]**	1.9%	*ns*	0.7%	*ns*
CM has hit/assaulted or used weapon against someone	31.9%	28.2%	32.9%	*ns*	37.9%	1.49 [1.14, 1.96]**	32.3%	*ns*	29.0%	*ns*
CM has stolen property	1.2%	1.0%	1.7%	*ns*	1.1%	*ns*	1.0%	*ns*	1.7%	*ns*
CM is or was in gang	3.9%	2.1%	7.7%	3.86 [2.13, 7.02]***	5.5%	2.73 [1.55, 4.79]**	6.5%	3.15 [1.26, 7.87]^+^	5.4%	2.58 [1.03, 6.44]^+^
*Contact with police*										
CM questioned by police	16.6%	11.1%	24.3%	2.57 [1.80, 3.65]***	20.9%	2.08 [1.47, 2.93]***	18.1%	1.78 [1.16, 2.76]**	16.3%	1.66 [1.04, 2.64]^+^
CM given warning by police	9.3%	5.4%	15.0%	3.06 [1.93, 4.86]***	15.0%	3.02 [2.04, 4.49]***	6.7%	*ns*	11.1%	2.29 [1.39, 3.78]**
CM arrested by police	1.4%	0.6%	3.5%	5.82 [1.59, 21.36]**	1.8%	*ns*	1.6%	*ns*	0.7%	*ns*
Criminal victimization outcomes										
CM was violently assaulted/had weapon used on	23.8%	21.8%	28.3%	1.41 [1.06, 1.87]^+^	27.2%	*ns*	21.2%	*ns*	19.9%	*ns*
CM had something stolen	7.8%	6.6%	8.5%	*ns*	11.7%	1.87 [1.23, 2.84]**	6.4%	*ns*	6.5%	*ns*
CM was sexually assaulted	2.8%	2.5%	3.4%	*ns*	2.3%	*ns*	2.2%	*ns*	0.8%	.29 [.10, 86]^+^
Mental health outcomes										
Past-year self-harm	15.4%	13.0%	16.8%	*ns*	19.9%	1.81 [1.28, 2.55]**	16.3%	*ns*	15.4%	*ns*
MFQ depression symptoms	5.71 (.08)	5.22 (.10)	6.46 (.46)	.04 [.02, .06]**	6.82 (.37)	.05 [.03, .07]***	5.53 (.38)	*ns*	6.19 (.51)	*ns*
Rosenberg self-esteem	10.49 (.05)	10.71 (.06)	10.24 (.16)	–.07 [–.11, –.03]**	10.05 (.18)	–.10 [–.15, –.05]***	10.49 (.22)	*ns*	10.05 (.19)	–.07 [–.12, –.02]**

^+^*p* < .05. **p < .01. ***p < .001.

In general, we found more increased risks (in comparison to the stable *two-parent/economic advantage* class) when the transition away from low risk occurred between the 9-month and 3-year assessment.

#### Transition from the “two-parent/economic advantage” to the “two-parent/high-conflict” class by age 3

##### Risk behavior outcomes

There were no increased or decreased risk behavior outcomes in adolescents who transitioned to the *two-parent/high-conflict* class by age 3.

##### Criminal behavior outcomes

Adolescents who transitioned to the *two-parent/high-conflict* class by age 3 were significantly more likely to carry a weapon and be affiliated with a gang. These adolescents were also more likely to be questioned, cautioned, or warned and arrested by the police.

##### Criminal victimization outcomes

There were no increased or decreased criminal victimization outcomes in adolescents who transitioned to the *two-parent/high-conflict* class by age 3.

##### Mental health outcomes

Although there was no significant difference in self-harm, adolescents whose families transitioned into the *two-parent/high-conflict* class at age 3 had significantly higher depression and lower self-esteem.

#### Transition from “two-parent/economic advantage” to “single-parent/economic hardship” class by age 3

##### Risk behavior outcomes

Adolescents who had transitioned to the *single-parent/economic hardship* class between 9 months and 3 years of age were more likely to binge drink and use illegal drugs compared to their peers who remained in the stable *two-parent/economic advantage* group.

##### Criminal behavior outcomes

Adolescents whose families had transition to the *single-parent/economic hardship* class before age 3 were more likely to carry a weapon, assault or use a weapon against someone, and have an affiliation with a gang. These adolescents were also significantly more likely to be questioned by the police and have been given a warning or caution by the police.

##### Criminal victimization outcomes

Adolescents who transitioned to the *single-parent/economic hardship* class at age 3 were significantly more likely to have something stolen from them.

##### Mental health outcomes

There was a significantly increased rate of self-harm, depression, and reduced self-esteem in adolescents whose families had transitioned to the *single-parent/economic hardship* class by age 3.

#### Transition from “two-parent/economic advantage” to “two-parent/high-conflict” class between age 3 and 5

##### Risk behavior outcomes

There were no significantly increased or decreased risk behavior outcomes in adolescents who transitioned into the *two-parent/high-conflict* class between age 3 and 5, compared to those who remained stable in the low-risk class.

##### Criminal behavior outcomes

Adolescents who transitioned to the *two-parent/high-conflict* class between age 3 and 5 were significantly more likely to vandalize or damage property and be questioned by the police.

##### Criminal victimization outcomes

There were no significantly increased criminal victimization outcomes for adolescents who transitioned to the *two-parent/high-conflict* class between age 3 and 5.

##### Mental health outcomes

There were no significantly increased poor mental health outcomes for adolescents who transitioned to the *two-parent/high-conflict* class between age 3 and 5.

#### Transition from “two-parent/economic advantage” to “single-parent/economic hardship” class between age 3 and 5

##### Risk behavior outcomes

There were no significant differences in risk behavior outcomes for adolescents who had transitioned to the *single-parent/economic hardship* class between 3 and 5 years of age, compared to those who remained stable in the low-risk class.

##### Antisocial and criminal behavior outcomes

Adolescents who transitioned to the *single-parent/economic hardship* class between age 3 and 5 were significantly more likely to be cautioned or receive a warning from the police.

##### Criminal victimization outcomes

There were no differences in criminal victimization outcomes for adolescents who had transitioned to the *single-parent/economic hardship* class between 3 and 5 years of age.

##### Mental health outcomes

There was reduced self-esteem in adolescents whose families had transitioned to the *single-parent/economic hardship* class between age 3 and 5 than those in the stable *two-parent/economic advantage* class.

#### Transitions between classes: Transition out of “single-parent/economic hardship” (high-adversity) class

We next looked at how families transitioned away from the *single-parent/economic hardship* class. We examined transitions to the low-adversity *two-parent/economic advantage* class at ages 3 and 5. There were insufficient numbers transitioning to the *two-caregiver/economic hardship* or *two-parent/high-conflict class* at age 3 or 5. The comparison group was stable membership in the high-adversity *single-parent/economic hardship* class. See Table 7 for a summary of these transitions and full results.

**Table 7.** Risk behavior, criminal behavior, victimization, and mental health outcomes for the entire sample, consistent “single-parent/economic hardship” families and movement away from consistent “single-parent/economic hardship” families (n = families with data at age 14)

	Overall	Stable single-parent/ econ. hardship (*n* = 638)	Single-parent → Low-risk two-parent/econ. adv. at age 3 (*n* = 289)		Single-parent → Low-risk two-parent/econ. adv. at age 5 (*n* = 102)	
Risk behavior outcomes	%	%	%	RRR/Coef. & CI	%	RRR/Coef. & CI
*Early sexualization*						
Oral sex/intercourse	3.4%	4.3%	3.7%	*ns*	2.9%	*ns*
*Substance use*						
Ever binge drinking	10.8%	11.8%	14.1%	*ns*	12.5%	*ns*
Cannabis/illegal drug use	5.7%	9.3%	5.5%	*ns*	9.5%	*ns*
Antisocial and criminal behavior outcomes						
*Antisocial behavior*						
Cohort member (CM) vandalized/damaged property	5.4%	8.7%	7.6%	*ns*	4.0%	*ns*
CM shoplifted	3.7%	6.0%	3.2%	*ns*	1.2%	.19 [.04, .81]^+^
Hacking	4.7%	6.2%	4.8%	*ns*	6.2%	*ns*
*Criminal activity*						
CM had carried weapon	2.7%	4.6%	1.7%	*ns*	1.9%	*ns*
CM has hit/assaulted or used weapon against someone	31.9%	33.4%	41.5%	*ns*	28.1%	*ns*
CM has stolen property	1.2%	2.4%	1.3%	*ns*	0%	—
CM is or was in gang	3.9%	7.9%	5.9%	*ns*	7.1%	*ns*
*Contact with police*						
CM questioned by police	16.6%	23.2%	24.3%	*ns*	21.6%	*ns*
CM given warning by police	9.3%	15.4%	16.1%	*ns*	12.5%	*ns*
CM arrested by Police	1.4%	1.8%	1.3%	*ns*	0%	—
Criminal victimization outcomes						
CM was violently assaulted/had weapon used on	23.8%	26.7%	25.6%	*ns*	27.0%	*ns*
CM had something stolen	7.8%	8.6%	12.3%	*ns*	7.8%	*ns*
CM was sexually assaulted	2.8%	3.6%	1.4%	*ns*	0.8%	*ns*
Mental health outcomes						
Past-year self-harm	15.4%	18.5%	10.4%	.51 [.31, .85]^+^	20.5%	*ns*
MFQ depression symptoms	5.71 (.08)	6.29 (.37)	5.23 (.45)	*ns*	5.12 (.92)	*ns*
Rosenberg self-esteem	10.49 (.05)	10.00 (.16)	11.11 (.26)	.13 [.06, .20]***	10.69 (.50)	*ns*

^+^*p* < .05. **p < .01. ***p < .001.

#### Transition from “single-parent/economic hardship” to “two-parent/economic advantage” class at age 3

Adolescents whose family transitioned from a *single-parent/economic hardship* household to a low-adversity environment with better economic advantages (*two-parent/economic advantage*) by age 3 had significantly higher self-esteem, compared to those who remained stable in the high-adversity *single-parent/economic hardship class*. There was no significant difference for other outcomes (see Table 7 for marginal effects).

#### Transition from “single-parent/economic hardship” to “two-parent/economic advantage” class at age 5

There were no significantly increased or decreased rates of risk behavior, criminal victimization, or mental health outcomes associated with the transition to *two-parent/economic advantage* from the *single-parent/economic hardship* households at age 5.

#### Transitions between classes: Transition out of “two-parent/high-conflict” or “two-caregiver/economic hardship” (high-adversity) classes to “two-parent/economic advantage” (low-adversity) class

There was insufficient stability (i.e., no stable *two-parent high-conflict group* available) in order to compare the transition from either the *two-parent/high-conflict* class or the *two-caregiver/economic hardship class* to the *two-parent/economic advantage* class.

## Discussion

This study used latent class analysis with data from the UK Millennium Cohort study to identify latent subgroups with differing family adversity profiles, and patterns of stability and transition between these subgroups, over the preschool years (9 months, 3 years, and 5 years). We examined both whether levels of early adversity in the preschool years (based on class membership) was associated with adolescent mental health and risk behaviors at 14 years of age, and whether transitioning from lower to higher risk groups (and vice versa) would predict different outcomes. Transitioning away from or toward increased adversity may have differential long-term impacts, making this paper a useful step forward to looking at whether there is a developmentally sensitive early childhood period where children are more at risk of longer term outcomes when exposed to less than optimal environmental exposures. This may help inform suitable strategies for providing intervention and/or additional support to best help reduce the long-term consequences of early child adversity.

Overall, our findings indicate that it is possible to identify theoretically meaningful and statistically valid groupings/profiles of children based on preschool family adversity factors. In this case, drawing on available data in the Millennium Cohort, groups were categorized based on parent status and biological relationship to child, suboptimal parenting, conflict and use of force by partner, homelessness and poverty, and maternal or main caregiver treatment for depression. Four groupings of preschool family adversity profiles were identified across the 9-month, 3-year, and 5-year data collection points. The differences between these subgroups were primarily driven by parental status (single parent, two biological parents/caregivers) and levels of economic (dis)advantage, followed by increased parental conflict. The most prevalent class was the low-adversity *two-parent/economic advantage* class (76% at 9 months), followed by the high-adversity *single-parent/economic hardship* class (18% at 9 months). A smaller number of cohort members were in two higher adversity classes: the *two-parent/high-conflict* class (5%) and the *two-caregiver/economic hardship* class (2%) at 9 months. The vast majority of the sample (72%) remained in the same class across the three time points (i.e., remained stable across the first 5 years of life). However, this was mainly driven by two classes: the low-adversity *two-parent/economic advantage* and *high-adversity single-parent/economic hardship* classes. This relative stability of membership of family adversity classes is higher than that previously found (55%) by Dunn et al. (2011), although it should be noted that this previous study explored class membership over a longer period of time. The notable stability in family profiles observed over the preschool years highlights the importance of identifying or developing early interventions that take into account and target persistent or ongoing family adversity factors.

Supporting the classification of early family adversity, we found that compared to the low-risk comparison group (two biological parents with economic advantage), adolescents in the groups categorized by higher rates of economic disadvantage demonstrated a range of poor outcomes by adolescence. Specifically, those who were in the *single-parent/economic hardship* group were at increased risk of a range of poor outcomes at 14 years of age, including illegal drug use, criminal or antisocial behavior, as well as higher rates of self-harm and depression and lower self-esteem. Marginal effects were also found for an increased risk of being a victim of assault, although this difference did not reach the conservative significance level. Similarly, those in the higher risk economic disadvantage group, where there were two caregivers coupled with economic hardship, were also more likely to engage in risk-taking behavior (e.g., binge drinking, drug use, and criminality), to be a victim of a crime (assault), and had higher rates of depression and self-harm, and lower self-esteem, compared to those in the low-adversity economic advantage group. Thus, it is not only the lack of a second parent but the lack of economic resources that appears to drive such risk.

These findings compliment and extend on previous work showing early adversity can have significant consequences on young people's later mental health and well-being (Dunn et al., 2011; Masarik & Conger, 2017; St Clair et al., 2014). The impact of economic disadvantage and poverty on outcomes across the life span has been well documented (e.g., Flouri, Midouhas, & Joshi, 2014; Wagmiller, Lennon, Kuang, Alberti, & Aber, 2006). This includes evidence that children born to low socioeconomic status families are three times more likely to engage in criminal activities than those born to high socioeconomic status families (Fergusson, Swain-Campbell, & Horwood, 2004) and that the amount of time spent in poverty in early childhood is associated with later increased internalizing and externalizing problems (Evans & Cassells, 2014). Drawing on UK cohort data, our own findings reflect the broad impact that socioeconomic disadvantage can have on young people's outcomes, across mental health and functional domains, including being both a victim and a perpetrator of criminal behavior. There are various processes by which early economic disadvantage may lead to a range of poor long-term outcomes for young people. For example, in houses categorized by high rates of economic deprivation, it is plausible that the parent may have fewer emotional and physical/financial resources to provide the same level of emotional support and physical supervision of the child that may be provided in houses with two parents and economic stability. This may be particularly the case for single-parents living with economic adversity, where emotional and physical resources are potentially even further stretched. More broadly, living in less safe neighborhoods, increased household stress, educational underachievement, and exposure to criminal or antisocial behavior (particularly through peer groups) have all been identified as potential processes that may link socioeconomic disadvantage to poor outcomes (e.g., Evans & Cassells, 2014; Fergusson et al., 2004). Overall, our findings show that living with early economic disadvantage is associated with adolescent outcomes that not only represent a personal cost to the individual but also likely represent a substantial societal and economic cost, including via involvement with police, loss of productivity, and access to services (for increased psychological difficulties).

We were also able to examine adolescent outcomes associated with being a member of a class characterized by high rates of parental conflict and force. While this class also had elevated rates of poverty (although not as high as the previously discussed classes), it was primarily categorized by high rates of parental conflict, poor early attachment, and high rates of maternal depression. Compared to those in the low-adversity class (low conflict and economic advantage), the adolescents in this higher risk group were more likely to engage in binge drinking and drug use, along with criminal and antisocial behaviors (carrying a weapon, gang membership, vandalizing and damaging property, and being questioned by police). Criminal and antisocial outcomes were particularly pronounced for males. They also had higher rates of self-harm and reduced self-esteem, with some evidence that this group were also more likely to be the victim of sexual assault in adolescence. There was also some evidence of a gender interaction for these outcomes, with girls in the higher adversity class having particularly elevated rates of both being sexually assaulted and perpetrating assault, compared to girls in the low-adversity class. While this class was small and less stable than the *two-parent/economic advantage* and *single-parent/economic hardship* classes, the pattern of results adds to growing literature showing the potential negative consequences of children's exposure to violence in the home, including a meta-analytic review that showed exposure to domestic violence was moderately associated with children's internalizing and externalizing problems (Evans, Davies, & DiLillo, 2008). Again, there are various pathways that may link early exposure to family violence and poor outcomes, including the impact on the parent–child relationship and potential consequences of poorer maternal mental health on child outcomes (Goodman et al., 2011), both areas associated with poorer child outcomes and factors that were also elevated in this class.

As a secondary aim, we explored whether transitions either from lower to higher risk classes, or vice versa, may predict different outcomes by adolescence. The purpose of such analyses were to explore whether there may be early developmentally sensitive periods for exposure to family adversity. A small proportion of cohort members in the low-adversity two-parent with economic advantage class at 9 months made a transition away from this low-risk group, characterized by a relationship breakdown and less economic resources (7% prior to age 3, 5% between 3 and 5 years) or by a move to the higher risk *two-parent/high-conflict* class (5% prior to age 3, 6% between 3 and 5 years). Overall, our findings on these lower to higher risk transitions indicated that increased risks, in terms of adolescent risk behaviors and mental health, were largely associated with transitions to higher risk groups occurring between 9 months and 3 years, rather than between 3 and 5 years old. Compared to adolescents who remained in the low-risk *two-parent economic advantage* group, those who experienced a breakdown in their parents’ relationship prior to 3 years old (resulting in a move to the *single-parent/economic hardship* class) were at increased risk of binge drinking and illegal drug use, along with criminal behavior (carrying a weapon, assault, and gang membership), being questioned or cautioned by the police, having something stolen, depressive symptoms, self-harm, and lower self-esteem. There were very few elevated risks observed (with the exception of contact with police and reduced self-esteem) when the transition from the low-risk group to the higher risk *single-parent/economic hardship* group happened between ages 3 and 5. Similarly, adolescents whose families had moved into the *two-parent/high-conflict* class prior to 3 years of age were more likely to engage in criminal behavior (use of a weapon and gang membership), have contact with the police, and have higher depressive symptoms and poorer self-esteem, compared to those who were in the stable low-risk class. Those adolescents whose families transitioned into a higher conflict environment between ages 3 and 5 showed increased risks for vandalizing property and being questioned by the police only.

These findings suggest that there may be a period of developmental sensitivity between 9 months and 3 years to the risk of losing a parent and having increased economic strain, or moving to a class categorized by increased domestic violence. It may be that a severity effect is in operation here. For example, for transitions to single parenthood with economic disadvantage, parental relationships that encounter the most serious difficulties may break down earlier in the child's life and may also be associated with more risk behaviors and emotional difficulties in adolescence. A further reason for the differences in outcomes for transitions before and after 3 years old age may be that the child is simply older when the parental relationship breaks down, and so the effective development of early attachment figures may serve an important protective function (Benoit, 2004; van IJzendoorn, Schuengel, & Bakermans-Kranenburg, 1999). Older children may also have additional sources of stability and support (e.g., the preschool environment). The implications of potential sensitive periods for specific adverse childhood experiences (i.e., marital/parental relationship breakdown) should also be considered in the context of the observed cumulative effect of adverse childhood experiences on later mental health and well-being (e.g., Evans et al., 2013; Hughes et al., 2016). Transitioning to a higher risk group earlier in development, by definition, means longer exposure to said adversity. Heterogeneity within the classes and associated potential differences in outcomes should also be acknowledged. Such complexity suggests a possible need to integrate population-based prevention with more targeted or individualized approaches to intervention—a challenging task.

We were also able to examine the impact of transitioning out of a higher risk class. Overall, there was less robust evidence of a positive or protective effect of transitioning from a high-adversity single-parent/economic hardship class to a low-adversity two-parent/economic advantage class. Adolescents who had transitioned to the low-adversity class before 3 years of age did have significantly higher self-esteem, and there were marginal effects for self-harm. In comparison, there was no evidence of any significant effect on outcomes if the transition occurred between 3 and 5 years old (i.e., where the adolescent was in the high-adversity class for longer). There is some evidence of the accumulative effect of growing up in a single-parent household on child outcomes. For example, a US cohort study found *always* living in a single-parent household placed a child at particular increased risk of poor outcomes, across behavioral and cognitive domains (Carlson & Corcoran, 2001). That said, the impact on single- versus two-parent households reduces substantially when accounting for factors such as maternal mental health and socioeconomic characteristics, and we were unable to compare effects associated with transitioning between the higher adversity groups or from the two-parent higher adversity groups (i.e., two-caregivers/economic hardship or two-parent/high-conflict) to the low-adversity class. It is also worth noting that there was heterogeneity within groups. Even in the low-adversity group, there were a proportion (15%–18%) living in poverty. Some of the families who moved out of the single-parent/economic deprivation group may have actually still experienced ongoing economic deprivation, and the analyses are unable to explore finer grain questions around this heterogeneity. It was also only a small proportion who transitioned from the high- to the low-adversity group, again meaning conclusions are tentative and require replication. Regardless, our findings from the stable classes clearly show the negative impact of early adversity, particularly categorized by economic deprivation, on adolescent outcomes, across a range of domains.

### Strengths and limitations

This study has several strengths, including the use of cohort data to provide information on early family adversity for a large sample, without the need to rely on retrospective information, as well as the use of self-report in adolescence reducing the impact of single-informant bias (i.e., where the parent reports on adversity, their own mental health and the child's well-being). However, we also acknowledge several limitations, many of which are inherent to the use of cohort data. The measures used were largely based on single-item questions (in some cases collapsed into categorical measures) rather than standardized measures; thus, some associations could potentially have been underestimated. For example, to ensure we were able to use consistent data across time points, we necessarily had to use a dichotomous “seeking treatment” variable to define parent mental health, which is likely not to have captured the full spectrum of parent mental health and may have more specifically captured those parents with the most serious mental health needs. No data on childhood maltreatment or abuse was available, factors known to have strong relationships with later childhood outcomes (and thus ideally should have been accounted for; Chapman et al., 2004). We did include indicators of suboptimal parenting at each age, but this brought additional limitations in that there was not a consistent measure across all three time points. Rather, we measured poor attachment at 9 months and harsh discipline at 3 and 5 years. While not ideal, this does follow developmental trends for poor parenting to be indexed by attachment issues in the first year of life. Discipline should only begin after the first year, and so harsh discipline is an appropriate marker of suboptimal parenting only from the age 3 time point onward.

Overall, the family adversity factors of focus in the present study may best be characterized as “moderate” facets of family adversity, which is important to bear in mind when interpreting the findings. Significantly disadvantaged children (particularly looked-after children) were either excluded from the cohort study or may have been lost to follow-up in latter waves of data collection. Nevertheless, even with a specific focus on more moderate family adversity, we were able to identify stable classes of adversity that predicted a range of outcomes at 14 years of age. The resulting classes and patterns of transitions identified were meaningful in terms of the extant literature and statistically parsimonious. However, in some cases, small group numbers precluded statistical comparisons. As previously mentioned, there is also likely to be heterogeneity within groups. Our analyses also do not allow the exploration of finer grain questions within groups, which might explain differences in outcomes for class changes in the different developmental periods. For example, do those families with relatively more economic and other disadvantage compared to others within the same class experience parental relationship breakdown earlier in the child's life, and subsequent poorer outcomes? This remains an important area for future investigation. Finally, although the analysis benefits from prospective cohort data including information on a range of adolescent risk behaviors, our analysis does not take into account any changes in the sample occurring between the ages of 5 and 14 years, although family adversity markers are considered relatively stable during this age range (Dunn et al., 2011).

### Summary

We identified four stable classes of varying levels of preschool family adversity, with class differences largely driven by parental status (presence/absence of two parental figures) and economic disadvantage (shared by all but the low-risk class), followed by high parental conflict. Examining patterns of stability and transition between classes over time highlighted the relative stability of class membership between 9 months and 5 years for two classes: the low-adversity *two-parent/economic advantage* class and the high-adversity *single-parent/economic hardship* class. Less than a third of the sample moved into or away from adversity during the preschool years. Overall, our findings highlight the significant consequences of early family adversity on adolescent outcomes. Compared to adolescents who spent early childhood in two-parent households with economic advantages, those who were in groups characterized by factors such as high rates of poverty, parental conflict, and maternal depression, were at risk of a range of poor outcomes across mental health domains, as well as criminality and victimization. Such findings are important, as these outcomes in adolescence potentially act as a gateway for further poor outcomes across adulthood.

The findings have a number of implications when thinking about intervention. First, the ability to identify a number of stable early family adversity profiles, and their associated risks for later mental health and risk behavior, has the potential to guide early identification, prevention, and intervention. Second, the need for early identification and intervention focused on vulnerable subgroups is suggested by the particular risks associated with experiencing family breakdown prior to the age of 3, especially in the context of economic hardship. Third, given the overall stability of class membership in early childhood, interventions that take into account the continuity of family adversity factors (such as economic disadvantage and parental conflict) would be recommended. While parenting practices are generally considered more amenable to change (i.e., compared to socioeconomic status), and therefore an appropriate intervention target, our findings clearly show the negative long-term consequences of poverty also need to be acknowledged and addressed. More universal approaches to mitigating early childhood risk factors may be appropriate and (arguably) increasingly achievable in the context of increasing free child care provision in the United Kingdom. Fourth and finally, beyond early intervention and prevention, our findings show that young people who have experienced early family adversity, characterized by poverty, high parental conflict, and single parenthood, are at risk for poor outcomes in adolescence. Timely and effective psychological supports for these young people could assist in mitigating the further entrenchment of mental health difficulties and documented poor outcomes in adulthood.

## Supplementary material

For supplementary material accompanying this paper visit https://doi.org/10.1017/S0954579419000191. click here to view supplementary material
