# Supplementary material for: The influence of early familial adversity on adolescent risk behaviors and mental health: Stability and transition in family adversity profiles in a cohort sample
Source: Dev Psychopathol. 2020 May;32(2):437–54. doi: 10.1017/S0954579419000191 (PMC7525106; doi:10.1017/S0954579419000191)
Supplement: Supplementary file 1 [file S0954579419000191sup001.docx]

# Fit Indices for Latent Class Solutions

# Latent Class Transitions between 9 months, 3 years and 5 years

See Table S1 for the numbers and percentages of the total sample with each transitional pattern.

Table S1. Transitions between classes

| **Transition class** | **N** | **% of total sample** |
| --- | --- | --- |
| Classes 1-1-1 | 8,060 | 62.77% |
| Classes 1-1-2 | 39 | 0.30% |
| Classes 1-1-3 | 440 | 3.43% |
| Classes 1-1-4 | 380 | 2.96% |
| Classes 1-2-1 | 62 | 0.48% |
| Classes 1-2-2 | 26 | 0.20% |
| Classes 1-2-3 | 5 | 0.04% |
| Classes 1-2-4 | 13 | 0.10% |
| Classes 1-3-1 | 259 | 2.02% |
| Classes 1-3-2 | 7 | 0.05% |
| Classes 1-3-3 | 104 | 0.81% |
| Classes 1-3-4 | 88 | 0.69% |
| Classes 1-4-1 | 149 | 1.16% |
| Classes 1-4-2 | 33 | 0.26% |
| Classes 1-4-3 | 12 | 0.09% |
| Classes 1-4-4 | 455 | 3.54% |
| Classes 2-1-1 | 98 | 0.76% |
| Classes 2-1-2 | 2 | 0.02% |
| Classes 2-1-3 | 27 | 0.21% |
| Classes 2-1-4 | 9 | 0.07% |
| Classes 2-2-1 | 4 | 0.03% |
| Classes 2-2-2 | 7 | 0.05% |
| Classes 2-2-4 | 3 | 0.02% |
| Classes 2-3-1 | 18 | 0.14% |
| Classes 2-3-2 | 1 | 0.01% |
| Classes 2-3-3 | 18 | 0.14% |
| Classes 2-3-4 | 5 | 0.04% |
| Classes 2-4-1 | 5 | 0.04% |
| Classes 2-4-2 | 4 | 0.03% |
| Classes 2-4-3 | 1 | 0.01% |
| Classes 2-4-4 | 40 | 0.31% |
| Classes 3-1-1 | 273 | 2.13% |
| Classes 3-1-2 | 4 | 0.03% |
| Classes 3-1-3 | 66 | 0.51% |
| Classes 3-1-4 | 19 | 0.15% |
| Classes 3-2-1 | 4 | 0.03% |
| Classes 3-2-2 | 5 | 0.04% |
| Classes 3-2-3 | 1 | 0.01% |
| Classes 3-2-4 | 1 | 0.01% |
| Classes 3-3-1 | 38 | 0.30% |
| Classes 3-3-2 | 5 | 0.04% |
| Classes 3-3-3 | 76 | 0.59% |
| Classes 3-3-4 | 15 | 0.12% |
| Classes 3-4-1 | 14 | 0.11% |
| Classes 3-4-2 | 6 | 0.05% |
| Classes 3-4-3 | 3 | 0.02% |
| Classes 3-4-4 | 81 | 0.63% |
| Classes 4-1-1 | 270 | 2.10% |
| Classes 4-1-2 | 20 | 0.16% |
| Classes 4-1-3 | 32 | 0.25% |
| Classes 4-1-4 | 80 | 0.62% |
| Classes 4-2-1 | 20 | 0.16% |
| Classes 4-2-2 | 33 | 0.26% |
| Classes 4-2-3 | 2 | 0.02% |
| Classes 4-2-4 | 19 | 0.15% |
| Classes 4-3-1 | 28 | 0.22% |
| Classes 4-3-2 | 8 | 0.06% |
| Classes 4-3-3 | 23 | 0.18% |
| Classes 4-3-4 | 22 | 0.17% |
| Classes 4-4-1 | 148 | 1.15% |
| Classes 4-4-2 | 68 | 0.53% |
| Classes 4-4-3 | 17 | 0.13% |
| Classes 4-4-4 | 1065 | 8.29% |

# Additional Gender Interactions

As stated in the methods section, some gender interactions seemed be driven by no endorsement in one group. Due to the lack of variability, this often drove significant differences which even one endorsement would likely have eradicated. However, if there was a genuinely differing pattern we did include the interaction in the results section. The interaction results we excluded from the main paper are reported below for completeness.

**Economic disadvantage and high risk classes and adolescent outcomes**

**Economic Disadvantage Class.**

*Antisocial and criminal behaviour outcomes.* There was a gender interaction with stealing something and the economic disadvantage class at age 3, B = -12.65, 95%CI(-14.01,-11.28), *p* <.001. This was due to no rates of stealing in females who were in the economic disadvantage class at age 3, which was significantly lower than the rate of 1.4% in the low risk age 3 class, *p* <.001. There was no difference in the rate of stealing in boys in the age 3 economic disadvantage class, *p* = .08.

**Transition out of ‘low risk’ class**

**Transition to ‘Single Parent’ Class between Age 3 and 5**

*Criminal behaviour outcomes.* There was a gender interaction with police arrests and the transition to single parent from low risk environments between age 3 and 5, B = -12.79, 95%CI(-14.70,-10.88), *p* < .001. This was due to no endorsements in girls who transitioned to single parenthood by age 5, which was lower than the .2% found in the low risk group, *p* <.001. There was no difference within the boys who transitioned to a single parent household by age 5, *p* = .54.

**Transition out of ‘Single parent’ class.**

**Transition to the ‘Low Risk’ class at age 3.**

*Antisocial and criminal behaviour outcomes.* There was a gender interaction with arrests and transition from a single parent to a low risk environment at age 5, B = -14.31, 95%CI(-15.98,-12.65), *p* <.001. This was due to no endorsements in the girls who transitioned to a low risk environment at age 5, which was significantly lower than the 1.8% rate for those who remained in a stable single parent household, *p* <.001. There was no difference in the rate for boys who transitioned to a low risk environment (2.5%) when compared to boys in the stable single parent context (1.7%), *p* = .22.

**Transition to the ‘Low Risk’ class at age 5.**

*Antisocial and criminal behaviour outcomes.* There was a gender interaction with shoplifting and transition from a single parent to a low risk environment at age 5, B = -13.55, 95%CI(-15.23,-11.86), *p* <.001. This was due to no endorsements in the girls who transitioned to a low risk environment at age 5, which was significantly lower than the 6.4% rate for those who remained in a stable single parent household, *p* <.001. There was no difference in the rate for boys who transitioned to a low risk environment (2.2%) when compared to boys in the stable single parent context (5.6%), *p* = .61.

There was another significant interaction with contact with gang membership and transferring to a low risk environment, B = 15.86, 95%CI(14.11,17.61), *p* <.001. This was driven by a lack of endorsement for males who transferred to a high risk context, which was significantly lower than the 9% rate in the single parent context, *p* <.001. There was no difference in the rate for girls who transferred to a low risk environment (15.2%) when compared to girls who remained in a single parent environment (6.7%), *p* = .26.

*Criminal victimisation outcomes.* There was a gender interaction with sexual assault in those who transitioned out of the single parent class to the low risk class at age 5, B = 11.48, 95%CI(9.07,13.90), *p* <.001. This was due to no endorsement in boys who transitioned to a low risk context, which was significantly lower than the 1.8% rate found in the stable single parent class, *p* <.001. There was no difference in the rate for girls who transitioned and those who remained in the stable single parent class, *p* = .25.

**Transition Out of High Risk.**

**Transition to ‘single parent’ class by age 3.**

*Antisocial and criminal behaviour outcomes.* There was a gender interaction with stealing property and transferring to a ‘single parent’ household by the age of 3, B = -14.60, 95%CI(-17.32,-11.90), *p* <.001. This was due to no endorsement with females who transitioned to a ‘single parent’ household by age 3, which was significantly lower than the rate of 1.8% in girls in a ‘high risk’ environment at age 3, *p* < .001. There was no difference in stealing property in boys who transitioned to a ‘single parent’ household at age 3 (4.4%) compared to those in a ‘high risk’ environment at age 3 (0.6%), *p* =.11.

**Transition to ‘single parent’ class by age 5.**

*Antisocial and criminal behaviour outcomes.* There was a gender interaction with hacking and transfer to a single parent class from a high risk environment, B = 14.30, 95%CI(12.86,15.73), *p* <.001. This was due to no endorsement of hacking in males who transferred to a single parent household, which was significantly lower than the rate of hacking in a high risk environment at age 5 (8%), *p* < .001. There was no difference in the rate of girls who transferred to a single parent environment at age 5 (4.6%) when compared to those who were in a high risk environment at age 5 (5.6%), *p* = .76.
